# Retinal Neurovascular Impairment in Patients with Essential Hypertension: An Optical Coherence Tomography Angiography Study

**DOI:** 10.1167/iovs.61.8.42

**Published:** 2020-07-29

**Authors:** Qingsheng Peng, Yijun Hu, Manqing Huang, Ying Wu, Pingting Zhong, Xinran Dong, Qiaowei Wu, Baoyi Liu, Cong Li, Jinxian Xie, Yu Kuang, Danqing Yu, Honghua Yu, Xiaohong Yang

**Affiliations:** 1 Guangdong Eye Institute, Department of Ophthalmology, Guangdong Provincial People's Hospital, Guangdong Academy of Medical Sciences, School of Medicine, South China University of Technology, Guangzhou, China; 2 Shantou University Medical College, Shantou, China; 3 Aier School of Ophthalmology, Central South University, Changsha, China; 4 Aier Institute of Refractive Surgery, Refractive Surgery Centre, Guangzhou Aier Eye Hospital, Guangzhou, China; 5 Department of Cardiology, Guangdong Cardiovascular Institute, Guangdong Provincial People's Hospital, Guangdong Academy of Medical Sciences, School of Medicine, South China University of Technology, Guangzhou, China; 6 Southern Medical University, Guangzhou, China

**Keywords:** hypertensive retinopathy, optical coherence tomography angiography, retinal nerve fiber layer, home blood pressure monitoring

## Abstract

**Purpose:**

To investigate retinal neurovascular structural changes in patients with essential hypertension.

**Methods:**

This observational cross-sectional study consisted of 199 right eyes from 169 nondiabetic essential hypertensive patients, divided into groups as follows: group A, 113 patients with hypertensive retinopathy (HTNR); group B, 56 patients without HTNR; and a control group of 30 healthy subjects. Peripapillary retinal nerve fiber layer (RNFL), radial peripapillary segmented (RPC), ganglion cell–inner plexiform layer (GC-IPL), and superficial (SVP) and deep (DVP) vascular plexus density at the macula (6 × 6 mm^2^) were measured by optical coherence tomography angiography (OCTA).

**Results:**

DVP density was significantly reduced in groups A and B compared to the control group (group A DVP, *P* = 0.001; group B DVP *P* = 0.002). GC-IPL, RNFL thickness, and RPC and SVP density in group A were significantly decreased compared to the control group or group B (all *P* < 0.05). In hypertensive patients, GC-IPL and RNFL thickness were negatively correlated with severity of HTNR (GC-IPL, *r* = –0.331, *P* < 0.001; RNFL, *r* = –0.583, *P* < 0.001) and level of home blood pressure monitoring (HBPM) (GC-IPL, *r* = –0.160, *P* = 0.050; RNFL, *r* = –0.282, *P* = 0.001) and were positively correlated with SVP (GC-IPL, *r* = 0.267, *P* = 0.002; RNFL, *r* = 0.361, *P* < 0.001) and RPC density (GC-IPL, *r* = 0.298, *P* < 0.001; RNFL, *r* = 0.663, *P* < 0.001). Among subjects with grade 2 or 3 retinopathy, the superior RNFL was significantly thinner in patients with high HBPM level than in those with normal HBPM level (grade 2, *P* = 0.016; grade 3, *P* = 0.006).

**Conclusions:**

Reduction of retinal vessel density and RNFL thickness is observed in patients with HTNR and is inversely associated with level of HBPM.

Hypertension affects more than 40% of adults over the age of 25 years worldwide.^1^ Systemic complications, particularly cardiovascular complications, have been the main causes of mortality.^2^ In a survey of hypertension complications, 44% of the investigated population had been diagnosed with retinopathy.^3^ Previous studies have reported that subjects with hypertensive retinopathy (HTNR) had the same risk of cardiovascular disease mortality as patients with diabetes.^4^^,^^5^ It has been suggested that HTNR could serve as a valuable indicator of severe hypertension complications^6^ requiring more aggressive antihypertensive treatments.^7^^,^^8^ For this reason, fundus examinations are essential in patients with hypertension; in fact, in the 2018 guidelines of the European Society of Hypertension, fundoscopy examination was recommended for patients with grade 2 or grade 3 hypertension.^9^

Hypertension-mediated organ damage (HMOD) has been considered to be a strong risk factor for cardiovascular and cerebral diseases,^10^ as well as a marker of preclinical or asymptomatic cardiovascular diseases.^11^ Peripheral capillary rarefaction as evidence of HMOD can be observed in nailfold, conjunctival, and skin capillaroscopy or laser Doppler flowmetry.^12^ Because optical coherence tomography angiography (OCTA) provides robust microvasculature quantification, rarefaction can also be detected on the retina.^13^ Previous studies have reported a decrease in perfusion in the macular area along with ganglion cell–inner plexus layer (GC-IPL) thinning in essential hypertension.^14^^–^^16^ Perfusion reduction in the retina was also observed in hypertensive patients with poorly controlled blood pressure (BP).^17^ These early studies demonstrated that OCTA could be a supportive imaging method for the detection of HTNR and HMOD in hypertensive patients.^16^

Although decreased retinal perfusion in patients with hypertension has been previously investigated, there is little evidence regarding the change of perfusion in retinal vessels in the peripapillary area, which are believed to interact with the retinal nerve fiber layer (RNFL) as part of the cerebral neurovascular unit; whether or not the neurovascular impairment appears simultaneously is unknown.^18^ Also, few studies have considered the impact of different stages of HTNR on retinal OCTA features.^19^ Moreover, despite the fact that antihypertensive treatment has been proven to be an influence of retinal microcirculation,^20^ such effect was not considered in previous studies about retinal vessel density in patients with hypertension.^14^^–^^17^^,^^19^^,^^21^^,^^22^ Furthermore, the possible association between BP control and the OCTA neurovascular features in hypertensive patients had yet to be investigated. In this study, we conducted a prospective survey of patients with essential hypertension, aiming to discover the changes of retinal perfusion and thickness and their association with BP control in eyes with or without HTNR.

## Method

### Study Participants

This was a prospectively planned observational cross-sectional study that included a total of 199 right eyes from 169 hypertensive patients—group A, 113 patients diagnosed with HTNR; group B, 56 patients without retinopathy; 30 healthy controls—from the cardiology clinic of Guangdong Provincial People's Hospital in Guangzhou from January 2019 to January 2020. Subjects recruited were local Chinese patients between the ages of 40 and 70 years who had been diagnosed with essential hypertension. Excluded from the study were participants with moderate or severe refractive error (higher than +3.0 diopter or lower than –3.0 diopter), history of glaucoma, history of ophthalmic surgery or retinal diseases, secondary causes of hypertension, BP higher than 160/100 after treatment, HbA1c higher than 6.1%, cardiac functional classification higher than grade 1, hypertensive complications other than retinopathy, coronary heart disease,^23^ stroke history,^24^ neurodegenerative diseases,^25^ migraine,^26^ or sleep apnea.^27^ Patients with arrythmia or mental disorders or who were undergoing hormone therapies were also excluded for health and safety reasons. The study was approved by the ethics committee of Guangdong Provincial People's Hospital, Guangdong Academy of Medical Science, and was carried out in accordance to the tenets of the Declaration of Helsinki. Each participant was informed of the study and provided full consent in writing.

### Examination Procedures

Clinical records of the patients’ antihypertensive medications, blood tests, and office blood pressure measurements (OBPMs) were reviewed. Lifestyle risk factors and demographic data such as age, smoking history, drinking behavior, home blood pressure monitoring (HBPM) for 3 months, and psychological states were collected using interviewer-administered questionnaires. All patients’ OBPMs were taken before their participation using the same type of sphygmomanometer (HBP-9020; Omron, Kyoto, Japan) as in the clinic. Each OBPM was taken after a participant had been seated comfortably in the waiting room for 5 minutes and was recorded as the average of three consecutive measurements with a deviation of <10 mm Hg. Data for each patient's HBPM were collected through questionnaires every 2 weeks for the previous 3 months. All patients were taught to correctly operate the HBPM device (Omron 7130/7133/8102k) and were instructed to record their HBPM results each morning and evening. The cut-off HBPM level was set at 135/85 mm Hg (normal, HBPM < 135/85 mm Hg; high, HBPM ≥ 135/85 mm Hg). All patients who participated were taking antihypertension medications, including angiotensin channel enzyme inhibitor (ACEI)/angiotensin receptor blocker (ARB) monotherapy, calcium channel blocker (CCB) monotherapy, and low-dose ACEI/ARB and CCB combined therapy. The height of each subject was measured using a wall-mounted measuring tape, and weight was measured with a digital scale. Body mass index (BMI) was calculated using the formula proposed by Keys et al.^28^ (BMI = body weight [kg] ÷ height squared [m]). Smoking history was defined as those who had smoked in the past versus those who had not.

### Ocular Examinations

Each participant underwent visual acuity measurement, autorefractometry (HRK-7000A Auto Refractor; Huvitz, Inc., Anyang-si, South Korea), non-contact tonometry (TX-20 Full Auto Tonometer; Canon, Inc., Tokyo, Japan), slit-lamp examination, and fundus photography (TRC-NW8 non-mydriatic retinal camera, Topcon, Tokyo, Japan; D7500 DSLR camera, Nikon, Tokyo, Japan). Diagnosis and grading of HTNR were performed by two ophthalmologists specialized in retinal diseases using indirect fundoscopy and fundus photography; grading was based on Keith–Wagener–Barker and Mitchell–Wong classifications^6^ (κ = 0.883). Differing diagnoses were reassessed and diagnosed by a senior chief ophthalmologist. The arteriovenous ratio was manually calculated using Image J 1.51 (National Institutes of Health, Bethesda, MD, USA). The diameters of arterioles and venules 1.5 papillary diameters to the optic disc were measured 10 times to obtain an average. The arteriovenous ratio cutoff point for determining retinal arteriole narrowing was set at ½. The distribution of retinopathy grades in each group was listed in Table 1.

**Table 1.** Grading of Hypertensive Retinopathy in the Three Groups

	Retinopathy Grades		*n*		
Features	Mitchell–Wong	Keith–Wagener–Barker	With Retinopathy (*N* = 113)	Without Retinopathy (*N* = 56)	Normal Control (*N* = 30)
	—	0	0	56	30
Mild generalized retinal arteriolar narrowing	None	1	36	0	0
Definite focal narrowing and arteriovenous nipping	Mild	2	51	0	0
Signs of grade 2 retinopathy plus retinal hemorrhages, exudates, and cotton wool spots	Moderate	3	26	0	0
Severe grade 3 retinopathy plus papilledema	Severe	4	0	0	0

Keith-Wager-Barker and Mitchell-Wong are two grading systems for hypertensive retinopathy, the grading systems are mainly based on retinal fundoscopy and photography.

### Optical Coherence Tomography and Angiography

**Figure 1.**
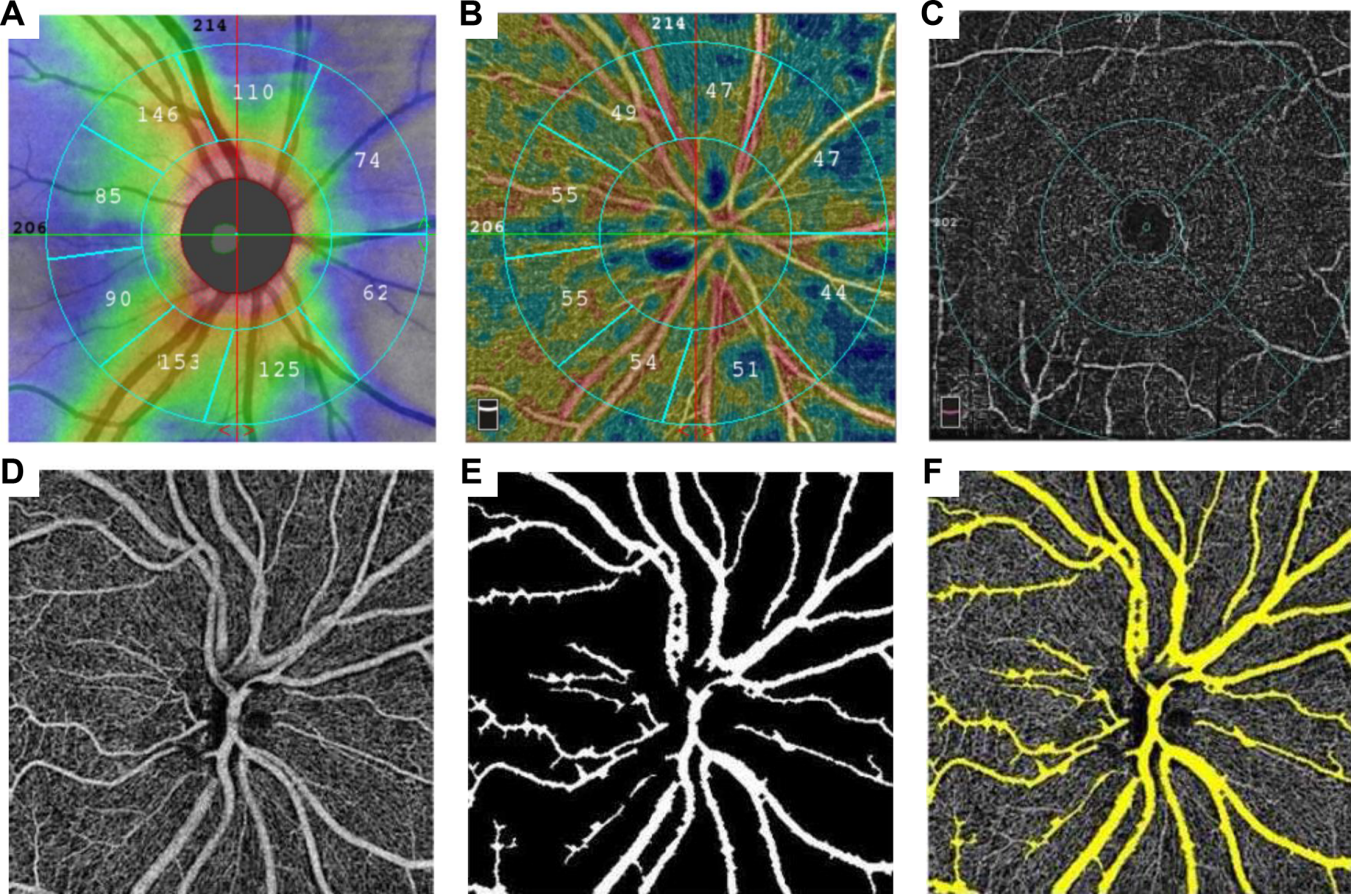
Layer identification and automatic segmentation using the AngioVue 2.0. (**A**) RNFL layer and its topographic map. (**B**) The 4.5 × 4.5-mm^2^ peripapillary area was divided into temporal, nasal, superior, and inferior quadrants, and each quadrant was then separated into two embedding areas. (**C**) A 6 × 6-mm^2^ macula area was split into segments based on distance to fovea; the parafoveal area was a concentric circle 1 mm to the fovea, and the perifoveal area was a concentric circle 1.5 mm to the parafovea. (**D**) The RPC layer was segmented into arteriovenous vessels (**E**) and capillaries (**F**). Vessel density was calculated separately.

**Figure 2.**
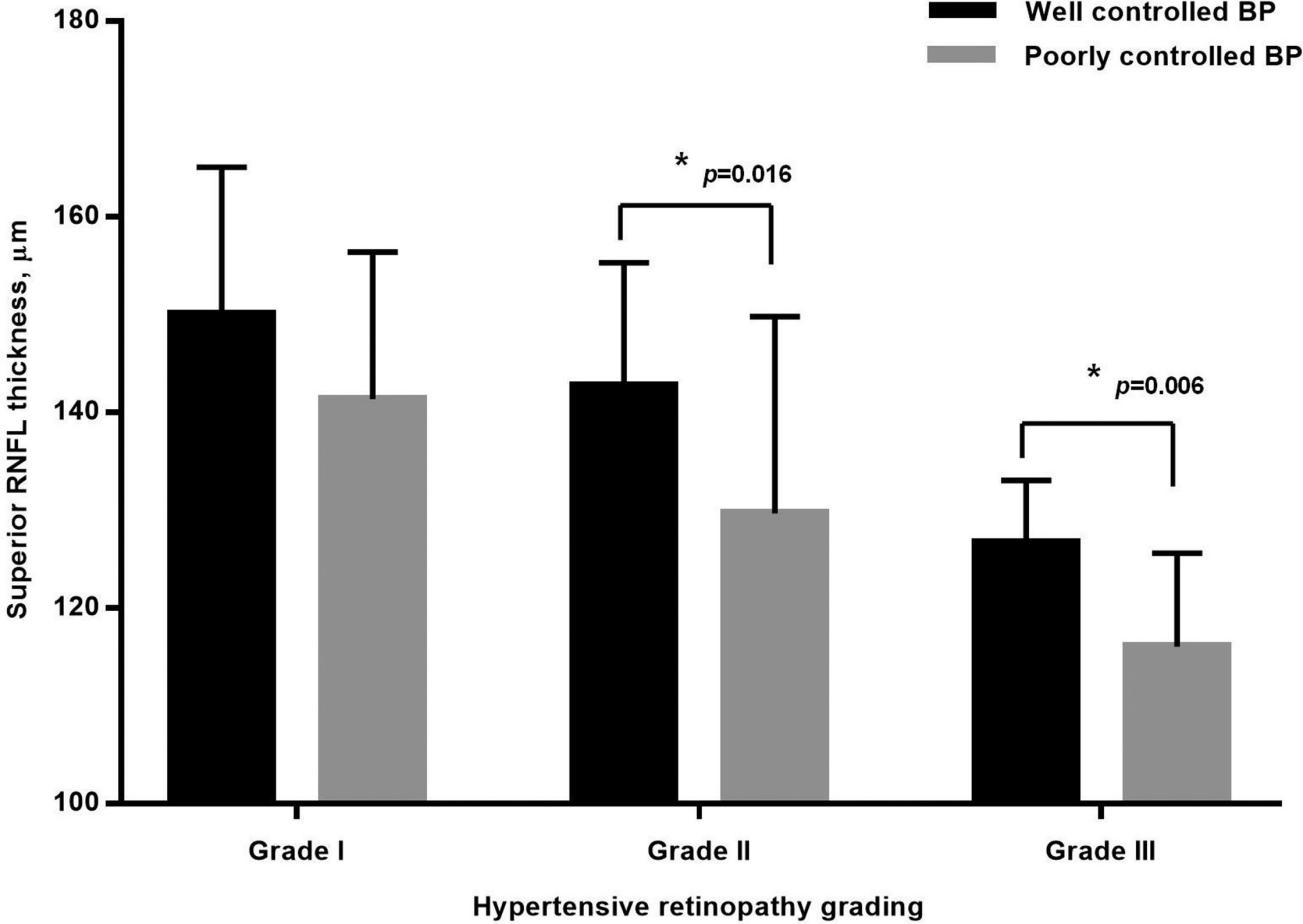
Superior RNFL thickness in patients with well-controlled or poorly controlled blood pressure in different grades of hypertensive retinopathy.

OCTA examination (RTVue-XR Avanti OCT System; Optovue, Inc., Fremont, CA, USA) with 6 × 6-mm^2^ macula HD scan and 4.5 × 4.5 mm^2^ disk HD scan mode (6 × 6-mm^2^ macula HD scan and disk HD scan mode are the high definition modes distinguished from the normal scan modes. Normal scans filmed with the RTVue-XR Avanti OCT System do not have the same image parameters described in the context.) was performed on each participant. The RTVue-XR Avanti uses split-spectrum amplitude-decorrelation angiography. The central wavelength of the system was set at 840 nm. The device can generate 304 × 304 scans in 2.9 seconds with a scanning speed at 70 kHz.^29^ The operating software of the RTVue-XR Avanti utilizes a projection-resolved OCTA algorithm to distinguish the superficial plexus; the RNFL and GC-IPL layers were automatically segmented. Parameters included RNFL and GC-IPL thickness and vessel density of the superficial vascular plexus (SVP), deep vascular plexus (DVP), and radial peripapillary capillaries (RPCs). Macular and peripapillary areas were further distinguished automatically, and capillary density was measured with a built-in feature called large-vessel masking, which removes vessels with a diameter of ≥3 pixels, approximately ≥33 µm for the 4.5-mm AngioVue optic disc (AngioDisc) scans (Figs. 1, 2).

### Extended Follow-Up for Exclusion

Because some information acquired in the study came from questionnaires, we set up an extended follow-up procedure. Patients with irregular clinical follow-up visits were asked to participate in a follow-up examination 1 month after completing the last questionnaire. In the follow-up confirmation process, five normal controls were excluded due to newly found hypertension, and 11 hypertensive patients were excluded because of changes in their antihypertensive treatment.

### Statistical Analysis

Normally distributed continuous variables were presented as mean ± SD; χ^2^ tests were used to compare nonparametric variables among the three groups. Independent *t*-tests were applied to compare the duration of hypertension between the two hypertensive groups. Tests for homogeneity of variance were applied to the OCTA data, including RNFL thickness, RPC density, and SVP and DVP density. ANOVA was used to compare the continuous variables. After baseline parameters showed no significance among the groups, multiple comparisons (Bonferroni) were carried out in post hoc analysis. After adjusting for age, BMI, spherical equivalence (SE), and duration of hypertension, analysis of covariance (ANCOVA) was used to investigate the impact of HTNR, various medication methods, and high HBPM levels on OCTA parameters among the hypertensive patients. Finally, partial correlation analyses were carried out to search for correlations between retinal vessel density and thickness and between OCTA parameters and HTNR grades (0, 1, 2, or 3) and HBPM levels, after adjusting for age, gender, hypertension duration, BMI, refractive error, smoking history, drinking behavior, and antihypertensive medications.

## Results

Details of the HTNR gradings are shown in Table 1. Demographic characteristics, antihypertension medications, and OCTA parameters of the three groups are shown in Table 2. ANCOVA of the OCTA parameters for the various antihypertensive therapies and stages of HTNR are shown in Table 3. Partial correlation analyses between OCTA and hypertension parameters are shown in Table 4. Best-corrected vision acuity (BCVA), SE, intraocular pressure (IOP), BMI, smoking history, and drinking behavior among the three groups were not significantly different (all *P* > 0.05). Antihypertension medications and hypertension duration between group A and group B were not significantly different (all *P* > 0.05)

**Table 2.** Demographics of the Three Groups and ANCOVA Results for OCTA Parameters

Demographic	With Retinopathy	Without Retinopathy	Normal Controls	*P* ^†^	*P* ^‡^	*P* ^§^	*P* ^||^	*P* ^¶^
Participants (*n*)	113	56	30	—	—	—	—	—
Females (*n*)	51	35	15	0.104	—	—	—	—
Smoking history (*n*)	10	6	3	0.924	—	—	—	—
Drinking behavior (*n*)	5	6	3	0.257	—	—	—	—
Normal HBPM level	59	30	30	<0.001^*^	—	—	—	—
ACEI/ARB	34	18	0	0.002^*^	—	0.785^#^	—	—
CCB	53	20	0	<0.001^*^	—	0.167^#^	—	—
ACEI/ARB and CCB	26	18	0	0.003^*^	—	0.203^#^	—	—
BCVA (logMAR), mean ± SD	–0.01 ± 0.06	–0.02 ± 0.05	—0.01 ± 0.03	—	0.872	1.000	1.000	1.000
RE (SE diopters), mean ± SD	–0.3 ± 2.2	–0.1 ± 1.9	0.1 ± 1.7	—	0.650	0.990	0.636	0.781
IOP (mm Hg), mean ± SD	16.3 ± 3.1	15.0 ± 3.8	14.7 ± 2.3	—	0.512	1.000	0.636	0.826
Age (y), mean ± SD	54.1 ± 11.2	52.4 ± 10.1	53.6 ± 9.2	—	0.759	0.816	1.000	1.000
HTN duration (y), mean ± SD	7.3 ± 8.0	6.6 ± 6.2	—	—	—	0.480^**^	—	—
BMI (kg/m^2^), mean ± SD	24.4 ± 3.3	24.8 ± 2.5	24.1 ± 1.9	—	0.470	1.000	1.000	0.712
OBPM (mm Hg), mean (IQR)								
Systolic BP	135 (130–149)	136 (127–147)	117 (110–122)	—	<0.001^*^	1.000	<0.001^*^	<0.001^*^
Diastolic BP	85 (80–95)	85 (79–93)	75 (65–81)	—	<0.001^*^	1.000	<0.001^*^	<0.001^*^
RNFL thickness (µm), mean ± SD								
Mean	110.3 ± 13.0	122.5 ± 7.5	123.3 ± 9.8	—	<0.001^*^	0.006^*^	0.001^*^	0.369
Superior	135.1 ± 19.8	150.7 ± 14.6	149.2 ± 12.6	—	<0.001^*^	0.005^*^	0.002^*^	0.677
Inferior	139.0 ± 21.3	159.0 ± 14.2	160.3 ± 13.3	—	<0.001^*^	0.006^*^	0.001^*^	0.468
Temporal	77.3 ± 11.2	83.4 ± 8.7	84.6 ± 10.6	—	<0.001^*^	0.001^*^	0.002^*^	1.000
Nasal	89.1 ± 15.2	95.5 ± 11.6	101.0 ± 16.6	—	<0.001^*^	0.022^*^	<0.001^*^	0.296
GC-IPL thickness (µm), mean ± SD	97.0 ± 6.7	101.2 ± 5.5	102.0 ± 6.9	—	<0.001^*^	<0.001	0.001^*^	1.000
RPC density (%), mean ± SD								
General	55.3 ± 2.8	56.8 ± 2.2	57.8 ± 1.8	—	<0.001^*^	0.001^*^	<0.001^*^	0.284
Capillary	48.9 ± 2.8	50.2 ± 2.0	50.8 ± 1.7	—	<0.001^*^	0.006^*^	0.001^*^	0.784
Superior hemifield	57.9 ± 4.0	59.7 ± 2.7	60.1 ± 2.0	—	0.001^*^	0.008^*^	0.007^*^	1.000
Inferior hemifield	56.8 ± 3.9	58.8 ± 2.3	59.6 ± 2.2	—	<0.001^*^	<0.001^*^	0.001^*^	0.982
Macular vessel density (%), mean ± SD								
Mean SVP	49.6 ± 3.9	50.2 ± 3.1	51.1 ± 3.6	—	0.001^*^	0.035^*^	0.002^*^	0.553
Parafoveal SVP	51.9 ± 4.8	52.7 ± 3.3	54.0 ± 3.8	—	0.002^*^	0.257	0.002^*^	0.178
Mean DVP	51.0 ± 5.6	51.8 ± 5.6	54.2 ± 5.5	—	0.001^*^	1.000	0.002^*^	0.001^*^
Parafoveal DVP	55.8 ± 3.5	54.8 ± 3.8	57.3 ± 4.2	—	0.005^*^	1.000	0.006^*^	0.011^*^

IQR, interquartile range; RE, refraction error; IOP, intraocular pressure.

* Statistically significant differences at *P* < 0.05.

† Nonparametric demographics of the three groups were analyzed using χ^2^ test.

‡ Continuous demographics and OCTA parameters of the three groups were analyzed using one-way ANOVA.

§ Obtained using post hoc Bonferroni correction between patients with retinopathy and without retinopathy.

|| Obtained using post hoc Bonferroni correction between patients with retinopathy and normal controls.

¶ Obtained using post hoc Bonferroni correction between patients without retinopathy and normal controls.

# Obtained using χ^2^ tests between patients with retinopathy and without retinopathy.

** Obtained using independent *t*-tests between patients with retinopathy and without retinopathy.

**Table 3.** ANCOVA Parameter Estimates of Antihypertension Medications and HTNR Grades

Reference	Comparing Group	Parameters	β	95% CI	*P*
ACEI/ARB & CCB	ACEI/ARB	SVP (%)	–1.569	–2.756 to –0.383	0.010^*^
		Parafoveal SVP (%)	–1.303	–2.867 to –0.433	0.012^*^
		DVP (%)	–1.731	–3.722 to 0.261	0.088
		Parafoveal DVP (%)	–0.527	–2.274 to 1.220	0.552
		General RPC (%)	–0.420	–1.492 to 0.652	0.440
		Segmented RPC (%)	–0.802	–1.898 to 0.206	0.151
		RNFL (µm)	1.907	–3.210 to 7.024	0.463
		GC-IPL (µm)	–0.695	–3.641 to 2.251	0.642
ACEI/ARB & CCB	CCB	SVP (%)	–1.201	–2.293 to –0.110	0.031^*^
		Parafoveal SVP (%)	–1.750	–3.068 to –0.433	0.010^*^
		DVP (%)	–2.360	–4.192 to –0.528	0.012^*^
		Parafoveal DVP (%)	–1.655	–3.127 to –0.184	0.028^*^
		General RPC (%)	–0.577	–1.564 to 0.409	0.250
		Segmented RPC (%)	–0.702	–1.610 to 0.206	0.129
		RNFL (µm)	0.585	–4.123 to 5.293	0.806
		GC-IPL (µm)	–3.545	–6.026 to –1.064	0.005^*^
HTNR grade 3	HTNR grade 2	RNFL (µm)	8.601	3.703 to 13.449	0.001^*^
		GC-IPL (µm)	3.010	0.159 to 6.179	0.048^*^
		SVP (%)	0.706	–0.505 to 2.287	0.209
		DVP (%)	0.272	–2.313 to 2.856	0.836
		General RPC (%)	0.092	–1.142 to 1.327	0.882
HTNR grade 3	HTNR grade 1	RNFL (µm)	17.275	11.780 to 22.770	<0.001^*^
		GC-IPL (µm)	5.030	1.475 to 8.586	0.006^*^
		SVP (%)	2.233	0.666 to 3.799	0.006^*^
		DVP (%)	1.430	–1.470 to 4.330	0.331
		General RPC (%)	2.030	0.645 to 3.415	0.004^*^

Multiple factors, including age, BMI, SE, and HTN duration, were adjusted in the model.

* Statistically significant differences at *P* < 0.05.

**Table 4.** Partial Correlation Results Between OCTA and Hypertension Parameters

	Correlated Parameters	*P*	Coefficient
HTNR grading	RNFL	<0.001^*^	–0.583
	Superior RNFL	<0.001^*^	–0.526
	Inferior RNFL	<0.001^*^	–0.535
	GC-IPL	<0.001^*^	–0.331
HBPM level	RNFL	0.001^*^	–0.282
	Superior RNFL	<0.001^*^	–0.315
	GC-IPL	0.050^*^	–0.160
	RPC	0.036^*^	–0.178
Mean RNFL thickness	RPC	<0.001^*^	0.663
	Segmented RPC	<0.001^*^	0.549
	SVP	<0.001^*^	0.361
	DVP	0.240	0.100
Superior RNFL thickness	RPC	<0.001^*^	0.554
	Segmented RPC	<0.001^*^	0.535
	SVP	0.002^*^	0.262
	DVP	0.854	0.016
GC-IPL thickness	RPC	<0.001^*^	0.298
	Segmented RPC	0.011^*^	0.221
	SVP	0.002^*^	0.267
	DVP	0.214	0.110
Systolic OBPM	RPC	0.535	0.054
	SVP	0.611	0.044
	DVP	0.037^*^	–0.238
	RNFL	0.217	–0.107
	GC-IPL	0.832	–0.018

* Statistically significant differences at *P* < 0.05.

### Comparison of SVP and DVP Vessel Density

Significant differences were found in both SVP (*P* = 0.001) and DVP (*P* = 0.001) vessel density among the three groups. In subgroup analysis, there were significant decreases of DVP vessel density in both group A (51.0 ± 5.6%, *P* = 0.002) and group B (51.8 ± 5.6%, *P* = 0.001) compared to normal controls (54.2 ± 5.5%). The parafoveal DVP were also significantly lower in both group A and group B compared to normal controls. SVP and parafoveal SVP were found to be significantly decreased only in group A compared to the control group (SVP, *P* = 0.001; parafoveal SVP, *P* = 0.011). None of the vessel density parameters of the macula differed significantly among the different grades of HTNR (all *P* > 0.05).

### Comparison of RPC Vessel Density

Significant differences were found in general, capillary, superior hemifield, and inferior hemifield RPC density among the three groups (general, *P* < 0.001; capillary, *P* < 0.001; superior hemifield, *P* = 0.001; inferior hemifield, *P* < 0.001). In the subgroup analysis, the general, capillary, superior hemifield, and inferior hemifield RPC densities in group A were significantly lower than in both group B (general, *P* *=* 0.001; capillary, *P* = 0.006; superior hemifield, *P* = 0.008; inferior hemifield, *P* < 0.001) and the control group (general, *P* *<* 0.001; capillary, *P* = 0.001; superior hemifield, *P* = 0.007; inferior hemifield, *P* = 0.001). No significance in RPC vessel density was found between group B and the control group (all *P* > 0.05).

### Comparison of RNFL and GC-IPL Thickness

There were significant differences in mean, superior, inferior, temporal, and nasal RNFL and GC-IPL thickness among the three groups (all *P* < 0.05). In the subgroup analysis, greater RNFL thinning was found in group A compared to normal controls in all of the sectors (all *P* < 0.05) or compared to group B (all *P* < 0.05). Greater GC-IPL thinning was also found in group A compared to both group B (*P* < 0.001) and the control group (*P* = 0.001).

In patients with HTNR, the RNFL thinning observed was most significant in the superior quadrant in patients with grade 2 HTNR (high HBPM, 129.7 ± 20.1 µm; normal HBPM, 142.8 ± 12.5 µm; *P* = 0.015) or grade 3 HTNR (high HBPM, 116.1 ± 9.5 µm; normal HBPM, 125.8 ± 6.3 µm; *P* = 0.006) in combination with high HBPM (Fig. 2).

### ANCOVA of Antihypertensive Medications and HTNR Grades

In the ANCOVA model regarding all hypertensive patients, patients under monotherapies were shown to have lower mean SVP and parafoveal SVP vessel density than patients taking ACEI/ARB and CCB combined therapy (all β < 0; all *P* < 0.05) after adjusting for age, BMI, SE, and hypertension duration. Patients taking only CCB also had significantly lower mean DVP and parafoveal DVP density (all β < 0; all *P* < 0.05). General RPC, SVP density, RNFL, and GC-IPL thickness in patients with grade 3 retinopathy was significantly lower than in patients with grade 1 retinopathy (all β < 0; all *P* < 0.05). Among patients with different HTNR grades, the thinning of both RNFL and GC-IPL was more severe in patients with grade 2 or 3 HTNR compared to grade 1 HTNR (all *P* < 0.05). Details of the ANCOVA results are listed in Table 3.

### Correlation Analysis Between OCTA Parameters and Hypertension Data

In the partial correlation analysis, after adjusting for age, gender, BMI, hypertension duration, refractive error, smoking history, drinking behavior, and antihypertensive medications, the severity of HTNR was negatively correlated with mean RNFL thickness (*r* = –0.583, *P* < 0.001), superior RNFL thickness (*r* = –0.526, *P* < 0.001), inferior RNFL thickness (*r* = –0.535, *P* < 0.001), and GC-IPL thickness (*r* = –0.331, *P* < 0.001). Level of HBPM was negatively correlated with mean RNFL (*r* = –0.282, *P* = 0.001), superior RNFL (*r* = –0.315, *P* < 0.001), GC-IPL thickness (*r* = –0.160, *P* = 0.050), and RPC density (*r* = –0.178, *P* = 0.026). Mean superior RNFL and GC-IPL thickness was found to be positively correlated with RPC, segmented RPC, and SVP density (*r* = 0.221–0.663; all *P* < 0.05). Systolic OBPM was found to be corelated with DVP density (*r* = –0.238, *P* = 0.037). Details about the correlations are shown in Table 4.

## Discussion

Results of our prospective study showed that retinal vessel density was significantly reduced in patients with essential hypertension (with or without HTNR), but RNFL and GC-IPL thinning was only detected in patients with HTNR. We found that retinal capillary rarefaction in the DVP was observed in hypertensive patients, and its severity was independent of the presence of HTNR. This indicates that retinal hypoperfusion exists regardless of hypertensive retinal complication. It implies that retinal hypoperfusion occurs when hypertension has developed. More evidence from future studies is needed to fill in the gaps in our understanding of retinal perfusion change between the prehypertension state and hypertension. On the other hand, we observed RNFL and GC-IPL thinning only in patients with HTNR. Moreover, RNFL thickness was strongly correlated with RPC density. These findings suggest that hypertension causes both retinal vascular and neural damage, and it is very likely that RNFL thinning is preceded and caused by retinal peripapillary hypoperfusion in patients with essential hypertension.

A decrease in retinal vessel density has been reported by previous studies using OCTA.^14^^,^^15^^,^^17^ In the last decade, OCT studies have also confirmed RNFL thinning as a form of hypertensive retinal damage.^30^ Yu et al.^31^ found a close correlation between RPC volume and RNFL thickness. These reports suggest that retinal vessel rarefaction may be associated with neural damage in patients with essential hypertension. In our study, RPC rarefaction and GC-IPL and RNFL thinning were only detected in hypertensive patients with HTNR, whereas DVP rarefaction was found in both hypertensive groups. It has been speculated that DVP is a more sensitive indicator of vascular degeneration than SVP. Our findings support the notion that early variations of retinal hypertensive impairment begin with altered DVP density. The findings from patients with HTNR may also lead to exploring the theory that retinal neural damage occurs along with macular and peripapillary capillary rarefaction in hypertension populations. A similar hypothesis was also proposed in diabetic retinopathy in an OCTA study.^18^ Recently, it has been proposed that the neurovascular unit (NVU) could be a mechanism for stroke.^32^ The NVU theory would explain the high prevalence of ischemic stroke in hypertensive patients with HTNR and the fact that structural and functional changes induced by hypertension predispose an individual to ischemic stroke with damage to integral functions of the NVU,^33^ reflecting common pathophysiology between the two major vascular diseases of hypertension and stroke.^4^ Cerebral research has found that hypertension-evoked remodeling of cerebral vessels leads to hypoperfusion and exacerbates ischemia-driven pathways, disrupting the integrity of the blood–brain barrier and allowing infiltration of inflammatory cells and cytokines into the brain.^34^^,^^35^ Similar to cerebral NVU findings, RNFL thinning in patients with retinal cotton wool spots has been observed on retina photography.^36^ In the retina, an extension of cerebral tissue, damage to the NVU caused by hypertension could be visualized in vivo by OCTA, which would provide a fresh perspective for future research in stroke.

In a cross-sectional observation, Chua et al.^17^ compared hypertension patients who had well-controlled BP with those who had poorly controlled BP. The results showed that patients with poorly controlled BP had sparser retinal capillary density. Our study further exhibited the impact of BP control on OCTA parameters. The elevated daily BP caused both neural and vascular retinal impairments. Earlier longitudinal cohort OCT studies also showed similar results, reporting that prolonged hypertension had a significant impact on the neuroretina, reducing GC-IPL and RNFL thickness.^21^^,^^37^ A recent OCT study also found neurodegeneration in patients with only mildly elevated blood pressure independent of aging.^38^ Our study further corroborates the suggestion that major hypertension-mediated neural and vascular changes in the retina occur in both the peripapillary and macular areas. Furthermore, the true correlation between neurodegeneration and HBPM may be more complex than we think and may not be a linear one, as shown by traditional statistics. On the other hand, nonlinear models, such as deep clustering algorithms or radial basis function neural networks, may be more suitable tools for directly extracting useful information from retinal scan images and daily BP monitoring and using it to evaluate BP control and predict HMOD. Prediction of systolic BP with acceptable accuracy using retinal scan imaging via convolutional deep neural networks has been reported.^39^

To the best of our knowledge, these results for the first time demonstrate the significant effects of various types of antihypertensive therapy on retinal vessels. Activation of the renin–angiotensin system (RAS) is important in increasing peripheral resistance, which induces hypertension and plays a role in cerebrovascular disease.^40^ CCBs lower blood pressure mainly by reducing intracellular Ca^2+^ concentrations and relaxing peripheral blood vessels.^41^ One study reported the possible synergistic effect of CCB with ACEI/ARB on RAS. Combining ACEI/ARB with CCB neutralizes the side effects on the RAS and sympathetic nerve system due to CCB monotherapy; also, the possibility of peripheral edema during CCB monotherapy is reduced.^42^ On the other hand, the negative sodium balance caused by CCB could enhance the treatment effect of ACEI/ARB.^43^ Our results further our understanding of the peripheral perfusion improvement resulting from ACEI/ARB and CCB combined therapy. This study identified various effects of antihypertension medications on retinal vessel density, and future studies should further explore antihypertensive medications and retinal vasculature in patients with hypertension.

Based on the results of our study and previous studies, RNFL thinning could serve as an indicator of HTNR, which is considered important evidence of HMOD in clinical practice. Also, the reduction of retinal vessel density, especially DVP density, could be used as an indicator of pre-HTNR damage to the retina. Because GC-IPL and RNFL thickness and retinal vessel density can be accurately quantified by OCTA, OCTA features of hypertensive patients could be important references when setting a patient’s target blood pressure. Capillary rarefaction is a possible cause of retinal nerve fiber damage, so OCTA may become the examination of choice to detect retinal capillary rarefaction and determine whether intensified antihypertensive treatment should be applied. Furthermore, because RNFL thinning was more severe in the patients with higher grades of HTNR or poorly controlled BP, the need for more intensive antihypertensive treatment and setting lower blood pressure targets for these patients is evident. Moreover, OCTA examination should be recommended for HTNR patients for regular follow-up to keep track of retinal neurovascular impairments.

This study had some limitations. First, HBPM information was collected by questionnaire, and some patients might not have reported their actual HBPM status; for this reason, ambulatory blood pressure monitoring may be considered in future studies. Second, patients with grade 4 HTNR or relieved grade 4 HTNR were not included in the study due to the lacks of such patients in our clinic.

In conclusion, we found that retinal DVP rarefaction appears in the early stage of hypertension. Retinal perfusion and RNFL thickness at the peripapillary area are reduced in patients with hypertensive retinopathy after adjusting for antihypertensive treatments, and the retinal neurovascular impairment is associated with the level of blood pressure control.
